# Primary CNS Lymphomas: Challenges in Diagnosis and Monitoring

**DOI:** 10.1155/2018/3606970

**Published:** 2018-06-21

**Authors:** C. Chiavazza, A. Pellerino, F. Ferrio, A. Cistaro, R. Soffietti, R. Rudà

**Affiliations:** ^1^Department of Neuro-Oncology, University & City of Health & Science Hospital, Turin, Italy; ^2^Department of Neuro-Radiology, University & City of Health & Science Hospital, Turin, Italy; ^3^Positron Emission Tomography Center IRMET, Turin, Italy

## Abstract

Primary Central Nervous System Lymphoma (PCNSL) is a rare neoplasm that can involve brain, eye, leptomeninges, and rarely spinal cord. PCNSL lesions most typically enhance homogeneously on T1-weighted magnetic resonance imaging (MRI) and appear T2-hypointense, but high variability in MRI features is commonly encountered. Neurological symptoms and MRI findings may mimic high grade gliomas (HGGs), tumefactive demyelinating lesions (TDLs), or infectious and granulomatous diseases. Advanced MRI techniques (MR diffusion, spectroscopy, and perfusion) and metabolic imaging, such as Fluorodeoxyglucose Positron Emission Tomography (FDG-PET) or amino acid PET (usually employing methionine), may be useful in distinguishing these different entities and monitoring the disease course. Moreover, emerging data suggest a role for cerebrospinal fluid (CSF) markers in predicting prognosis and response to treatments. In this review, we will address the challenges in PCNSL diagnosis, assessment of response to treatments, and evaluation of potential neurotoxicity related to chemotherapy and radiotherapy.

## 1. Introduction

Primary Central Nervous System Lymphoma (PCNSL) is a rare extranodal non-Hodgkin lymphoma (NHL), which can involve brain, eye leptomeninges, and rarely spinal cord without evidence of systemic disease. PCNSL accounts for 1 to 3% of all NHL and about 3% of all primary brain tumours [1]. Approximately 95% of PCNSLs are diffuse large B-cell lymphomas, while the other 5% include T-cell, Burkitt, lymphoblastic, and marginal zone lymphomas [2]. Median age at disease presentation is 65 years, with a trend toward an increase of incidence in the oldest population [1, 3]. Risk factors for PCNSL include congenital and acquired immunosuppression (particularly HIV and posttransplant conditions).

Several pathogenetic mechanisms have been related to PCNSL, namely, mutations in specific genes (e.g., MYD88, PIM1, ATM, and TP53) and dysregulation in JAK/STAT, NF-kB, toll-like receptor, and B-cell receptor signaling pathways. PCNSL frequently shows 9p.24/PD-L1/PD-2 translocations and copy number alterations compared to other large B-cell lymphomas. Moreover, PCNSL has a selective angiotropism: the accumulation of tumor cells around blood vessels is thought to be the main cause of disruption of blood-brain barrier (BBB) [4]. From a pathological point of view, PCNSL is a “whole-brain disease” [5, 6], an important concept for both diagnosis and treatment.

In this review, we will discuss the challenges in the diagnosis and monitoring of PCNSL and also focus on baseline and long-term cognitive profile of PCSNL patients. 

## 2. Clinical Aspects

PCNSL presentation is commonly subacute with typical symptoms such as cognitive decline or personality changes, confusion, focal neurological deficits, headache, and/or nausea and vomiting due to intracranial hypertension. Sometimes rapid worsening of neurological status up to stupor can be seen. Seizures are less frequent in comparison to other brain tumors (10%-20% of patients) [7]. Leptomeningeal involvement is detected in 15-20% of cases [8], while symptoms (blurred vision and decreased acuity) due to ocular involvement (vitreous/retina) are seen in 30% and may precede or coexist with neurological symptoms [9].

Systemic B symptoms are unusual in patients with PCNSL.

Corticosteroids may induce a rapid improvement in clinical symptoms and radiographic features, and it is well known that at least 40% of the patients show steroid-induced responses. This point is important clinically but may be a disadvantage leading to false negative biopsies, thus delaying definitive diagnosis for months and even years. For this reason, until the diagnosis is established, corticosteroids should not be prescribed if there is a suspicion of PCNSL, unless severe worsening of patient's conditions occurs [4].

## 3. PCNSL Diagnosis: Role of Standard and Advanced MRI

**Figure 1:**
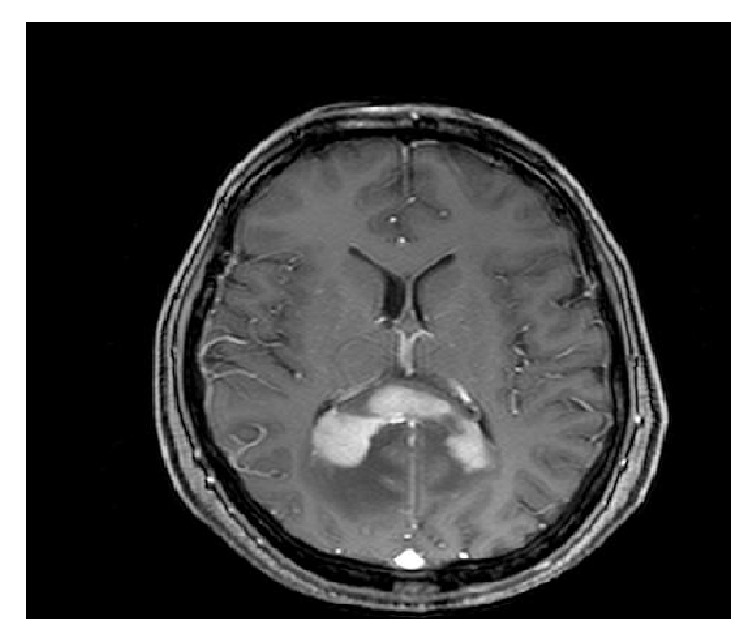
Contrast-enhanced axial T1-weighted MRI showing a PCNSL located in corpus callosum.

**Figure 2:**
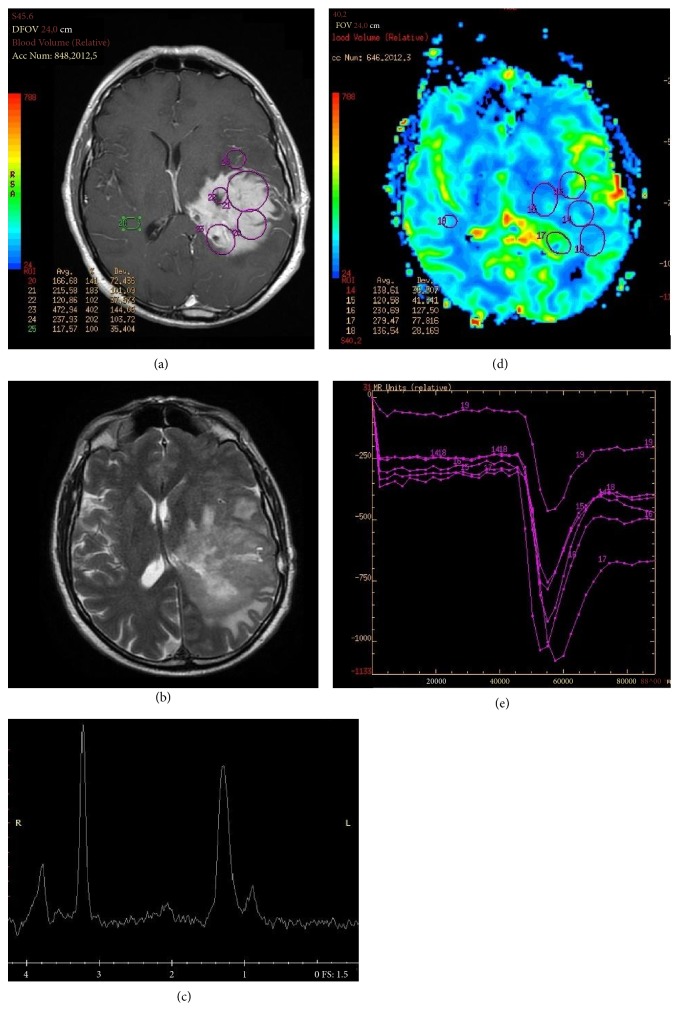
(a) Left temporoparietal PCNSL characterized by a homogeneous enhancing lesion on T1-weighted and (b) relatively low and inhomogeneous T2 signal on T2-weighted MRI. (c) Increased lipid peak on MRI spectroscopy and (d-e) increase of the regional cerebral blood volume (rCBV) when compared to the contralateral hemisphere.

Most common locations on MRI include periventricular white matter, basal ganglia, and corpus callosum [Figure 1], while cerebellum, brainstem, and spinal cord are less frequently involved [10]. In particular, spinal involvement is very uncommon and is seen in 3% of patients with PCNSL. The most common intramedullary localization is the cervical cord, with solitary lesions in the majority of patients [2, 10]. Due to the high cellularity, lesions are often hyperdense on CT and hypointense on T2-weighted MRI images with a variable amount of peritumoral edema [11, 12]. In most cases, the lesions enhance homogeneously [Figure 2(a)], but sometimes the enhancement is mild or with a ring pattern or is even absent [13]. Single lesions account for 70% of cases and multiple lesions for 30%: a multifocal presentation is more frequent in immunocompromised patients.

The differential diagnosis is with high grade gliomas (HGGs) and less often TDLs (tumefactive demyelinating lesions), metastases (MTS), and infectious and granulomatous diseases [14].

### 3.1. PCNSL versus HGG

The most significant MRI difference between PCNSL and HGG is the pattern of enhancement: homogeneous in PCNSLs and heterogeneous with necrotic areas in HGGs. Furthermore, optic tracts and basal ganglia infiltration are more frequent in PCNSL, while cerebral cortex is affected more often in HGGs; nonetheless, “superficial” PCNSL variants mimicking HGG can be found [15, 16].

Many studies have explored diffusion-weighted imaging (DWI) findings in PCNSL and gliomas [15, 17–24]. PCNSLs have higher cellularity and nuclear-cytoplasm ratio than glioblastomas (GBMs). Thus, in PCNSL, diffusion is more restricted with lower apparent diffusion coefficient (ADC) in comparison to GBM. GBMs have a solid portion, which can show a restricted diffusion, but their cystic-necrotic areas display high water molecules diffusion [15, 17–19]. Some authors have shown that the relative minimum apparent diffusion coefficient (rADCmin) is the most accurate parameter to differentiate PCNSL from GBM [22, 25, 26], and a cut-off value of 0.722 has been suggested, with 74.5% sensitivity and 74.1% specificity [26]. Another study [27] demonstrated that the initial area under the curve (IAUC) derived from dynamic contrast-enhanced MRI (DCE) may be a useful parameter together with ADC for differentiating PCNSL from atypical GBM (i.e., GBM with absent or limited necrosis), and thus the combination of quantitative ADC and permeability parameters from DCE MRI in tumor and peritumoral areas could help in discriminating PCNSL versus HGG [28].

^1^H-magnetic resonance spectroscopy (^1^H-MRS) can help in the differential diagnosis between PCNSL and GBM. Both tumors usually show reduced N-acetylaspartate (NAA) peaks (neuronal damage), elevated choline to creatine ratio (elevated membrane turnover), lipid peaks (due to release of fatty moieties by transformed lymphocytes in PCNSL and due to necrosis in GBM), and lactate peaks (anaerobic metabolism). An increase in lipid resonance in the absence of necrosis seems to be the most specific finding in PCNSL [29–31] [Figures 2(b)-2(c)]. Nonetheless, given the difficulty in excluding small necrotic areas in many GBMs, other peaks have been investigated, such as glutamate (Glu) and glycine (Gln) [32]: Aburano et al. [33] reported that PCNSL shows higher Glu/Cr and Glu/Glu+Gln ratios than GBM.

Jiang et al. [34] investigated the usefulness of amide proton transfer weighted studies (APTW) in the diagnosis of PCNSL. APT is a technique that detects endogenous mobile proteins and peptides in tissue, such as those dissolved in cytoplasm. PCNSL has a high nuclear-cytoplasm ratio and prominent nucleoli and a relative low concentration of mobile proteins. APTW imaging in PCNSL shows homogeneous hyperintensities in enhancing areas, while HGGs have quite heterogeneous APTW hyperintensities, more often larger than the Gd-T1 lesions.

PCNSLs lack abundant neovascularization [35]: thus, on dynamic susceptibility contrast-enhanced MRI (DSCE-MRI), they show higher regional cerebral blood volume (rCBV) than normal tissue but lower rCBV when compared to the GBMs [25, 36, 37] [Figures 2(d)-2(e)]. Blasel et al. [38] demonstrated in PCNSL a typical “shoulder-like” pattern of signal intensity dynamics in most cases. Another study [39] identified a threshold in the rCBV value (2.56), which could be used to differentiate PCNSL from HGG with >90% sensitivity and specificity and argued that the combination of rCBV and percentage of signal intensity recovery derived from DSCE-MRI could increase the accuracy of diagnosis.

#### 3.1.1. Take Home Message

In the daily clinical practice, in addition to MRI morphological features, DSCE-MRI and MRS are the most reliable tools for a differential diagnosis and should be performed in particular in elderly patients when a surgical approach (biopsy versus resection) is to be decided.

### 3.2. PCNSL versus TDL

Tumefactive demyelinating lesions are sometimes another challenging differential diagnosis, especially in younger patients. On MRI, TDLs are solitary lesions, with T2-FLAIR hyperintense appearance, larger than 2 cm with mass effect and/or edema and variable enhancement patterns (homogeneous or heterogeneous, nodular or diffuse, punctate, open, or closed rings) [40]. Symptoms at initial presentation include headache, cognitive impairment, seizures, confusion, and impaired state of consciousness, which are common in PCNSL patients as well. Nonetheless, TDL patients have a clinical and radiological response to steroid therapy similar to that observed in PCNSL patients.

For a noninvasive diagnosis of TDLs, advanced imaging techniques are widely used. The typical DWI feature of TDLs is the heterogeneity of ADC values [41]. Myelin destruction and vasogenic edema can cause ADC increase [30, 42], but TDLs can also show reduced ADC values due to infiltration of inflammatory cells, especially in the periphery of the lesions [43]. Overall, TDLs consist of hypocellular lesions with ADC values higher than PCNSL, which conversely shows lower, homogeneous ADC values given the high cellularity [26, 44].

On DSCE-MRI, rCBV values are higher in PCNSL than TDLs [44]. MRS findings can be similar in PCNSL and TDL, consisting in decreased NAA (neuronal damage in PCNSL/axonal injury in TDL), increased choline (proliferation in PCNSL/myelin breakdown in TDL), lipid peak (fatty moieties released by transformed lymphocytes in PCNSL/membrane breakdown in TDL), and/or lactate peak (anaerobic metabolism in both entities) [45–47].

PCNSL may rarely be preceded by “sentinel demyelination,” an entity characterized by histologically confirmed demyelinating inflammatory lesions, which mimics multiple sclerosis (MS) or acute disseminated encephalomyelitis (ADEM) [48–52]. It has been hypothesized that T-cell infiltrates, which can be found at biopsy, represent a cell-mediated immune response against the lymphoma, thereby masking the diagnosis of PCNSL [53, 54]. Another hypothesis is that steroids, administrated prior to brain biopsy, disrupt B-cell lymphoma cells, whereas activated T-cells may be relatively protected from steroid-induced apoptosis [54]. In this regard, a recent retrospective study examining approximately 1000 cases of PCNSL has reported that the effects of corticosteroids given before the biopsy rendered an accurate diagnosis difficult in up to 50% of cases [55].

Red flags suggesting PCNSL include advanced age, worsening of clinical conditions despite treatment for a demyelinating disease, and lesions progression on neuroimaging studies over time. Conversely, MS onset in old patients is often characterized by spinal cord involvement [49]. To improve the differential diagnosis between demyelinating diseases and PCNSL, tools such as visual evoked potentials, spinal cord MRI, and cerebrospinal fluid (CSF) examination for oligoclonal bands should always be performed. Moreover, especially in younger patients, a search in the past history for minor episodes of subacute visual or neurological deficits with spontaneous resolution, suggestive of a first MS relapse, should be performed.

#### 3.2.1. Take Home Message

In the daily clinical practice, this diagnostic problem is rare; however, when present, a combination of radiological, neurophysiological, and laboratory findings and the evaluation of their changes over time are needed.

### 3.3. PCNSL versus MTS

The typical localization of metastases includes the cortical gray-white matter junction of cerebral hemispheres, cerebellum, and basal ganglia in decreasing order. On MRI brain MTS can be solitary (40%) or multiple (60%), often with central necrosis, a peripheral ring of enhancement, and extensive edema [56–58]. On DWI, metastases show reduced diffusivity in the peripheral enhancing part and increased diffusivity in the central necrotic area and surrounding edema, while PCNSLs show a homogeneous reduced diffusivity due to the high cellularity [59]. rCBV is lower in PCNSL than metastases, due to lack of neovascularization. MRS studies investigating differences between PCNSL and metastases are not available.

#### 3.3.1. Take Home Message

In the daily clinical practice, when multiple lesions are evident on MRI, a differential diagnosis between brain metastases and PCNSL should always be taken in mind.

### 3.4. PCNSL versus Infectious and Granulomatous Diseases

In AIDS patients, a differential diagnosis between PCNSL and Toxoplasmosis Encephalitis (TE) is not always straightforward, since these two entities can show similar clinical findings and mass lesions on imaging. Clinically, patients with TE can present either acutely or insidiously with variable combination of headache, fever, encephalopathy, seizures, and focal deficits. On MRI, TE lesions are generally isointense to hypointense on T1-weighted images with ring or nodular enhancement, while in T2 they appear as hyperintense (during liquefactive necrosis), hypointense (in the posttreatment phase), or isointense, depending on the stage and composition of the abscess. They are often located in basal ganglia or in the junction between the white and gray matter of cerebral hemispheres and less commonly in the brainstem. Extensive T2-hyperintense vasogenic edema is typically present. Toxoplasma encephalitis lesions are multifocal on MRI in 86% of cases [60].

In DWI studies, TE lesions show higher ADC values than PCNSL [61]. The ability of MRS to differentiate TE from PCNSL depends on the voxel placement over necrotic or cellular areas and on the lesion stage. Nonetheless, in typical cases, lipid/lactate peaks (reflecting anaerobic, necrotizing inflammatory process) are present without elevated Cho/Cr ratio typical of PCNSL. Last, rCBV values are substantially higher in PCNSL as compared to TE lesions [62].

Neurosarcoidosis can present with multiple enhancing brain lesions mimicking PCNSL. Magnetic resonance imaging findings are variable. About 40% of patients with neurosarcoidosis have either leptomeningeal enhancement or multiple white matter enhancing lesions [63]. In these cases, differential diagnosis should be based on serum ACE levels, CSF examination (usually with pleocytosis, increased protein level, and oligoclonal bands), chest high-resolution CT (HRCT), and bronchoalveolar lavage (BAL). Nonetheless, biopsy of suspected granulomatous lesions is the gold standard for diagnosis [64].

#### 3.4.1. Take Home Message

In the daily clinical practice, this diagnostic problem is rare. However, patient history, CSF, and serum analysis are fundamental to exclude the hypothesis of infectious diseases. In particular CSF examination, ACE levels and HRTC are needed to exclude the hypothesis of neurosarcoidosis.

## 4. PCNSL Diagnosis: Role of PET

In 1992, Rosenfeld et al. reported a strong ^18^F-Fluorodeoxyglucose (18F-FDG) uptake in a group of 10 patients with PCNSL [65]. These characteristics depend on the high cellular density and increase glucose metabolism: the semiquantitative ^18^F-FDG uptake values, measured by maximum standardize uptake value (SUVmax), are reported to be 14–22 in PCNSL, and this value is about 2.5 times higher than the average SUV in the normal gray matter [66]. However, the high uptake of basal ganglia, cerebral cortex, and thalamus makes the diagnosis problematic.

^18^-FDG PET can play a role for diagnosis in patients who cannot undergo brain biopsy due to surgical risks, older age, or comorbidities. Yamaguchi et al. [67] demonstrated that inoperable PCNSL can indirectly be diagnosed with good accuracy on the basis of some PET-MRI criteria: extremely high tumor FDG uptake relative to normal gray matter and tumor reduction 1 week after corticosteroid administration on contrast-enhanced T1-weighted MRI.

Total body ^18^F-FDG-PET has an important role in PCNSL staging at diagnosis or in the follow-up, as it can diagnose a systemic disease with higher sensitivity than conventional imaging. Mohile et al. [68] showed that 7% of patients with suspected PCNSL were found to have systemic NHL by total body FDG-PET, while body CT scans and bone marrow biopsies were negative. Moreover, total body FDG-PET was positive in 27% of patients during restaging for recurrent disease.

Regarding amino acid PET, methionine (MET) uptake reflects an increase of amino acid transport and protein synthesis and is related to cellular proliferation. In the evaluation of brain lesions, MET has some advantages over FDG, stemming primarily from a low uptake in the normal brain. MET is thought to be useful for delineating tumor boundaries of PCNSL, with the area of increased uptake being larger than the enhancing lesions on MRI [69]. Kawase et al. [70] did not find significant differences between T/N (tumor to normal contralateral cortex activity) ratios on MET-PET and FDG-PET, although mean values of SUV on MET-PET in CNS lymphomas were significantly lower than those on FDG-PET. In this case series, 2 of 13 FDG-PET scans did not show a marked accumulation in the tumor (small disseminated lesions in one case and disseminated lesions overlaying the cortex in the other case), while increased MET uptake was observed in both patients.

Nonetheless, the experience in the use of amino acid PET in patients with PCNSL is still limited.

*Take Home Message*. The typical PCNSL metabolic pattern is an area of high homogeneous increase of ^18^F-FDG, more often in subcortical regions. Frequently, this area is more visible at visual analysis due to the presence of hypometabolism in the adjacent cortex because of compression or edema phenomena.

**Figure 3:**
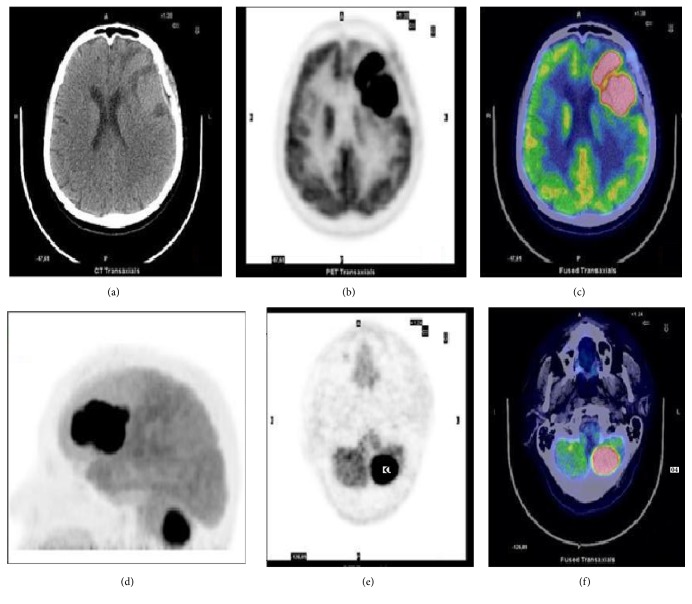
^18^F-FDG brain imaging in a 66-year-old woman with PCNSL. Axial CT (a), PET (b), and PET/CT fusion image (c) showing the FDG-avid lesion involving the left frontal lobe (SUVmax 42). (d) Maximum imaging projection (MIP), axial CT (e), and PET/CT fusion image (f) showing another lesion on the left cerebellar hemisphere.

These patterns are maintained in the disease recurrence as well [Figure 3].

### 4.1. PCNSL versus GBM or MTS

The ^18^F-FDG uptake in PCNSL is usually homogenous in contrast to the inhomogeneous uptake in GBMs and metastases. Kosaka et al. [71] identified maximum standard uptake values (SUVmax) on FDG-PET as the most important parameter for distinguishing lymphomas from other brain tumors. They used a SUVmax of 15 as a cut-off for diagnosing CNS lymphoma, and only one high grade glioma yielded a false-positive result. Based on these findings, Makino et al. [72] demonstrated that the accuracy of FDG-PET for differentiation of PCNSL versus GBMs and metastases was 0.86 when the SUVmax cut-off value was set at 12 with sensitivity of 100% and specificity of 71.4%.

Yamaguchi et al. [67] showed that FDG uptake using the T/N ratio was more reliable than SUVmax, since SUVmax is influenced by plasma glucose levels. The appropriate T/N ratio cut-off point was 2.0 for differentiating PCNSL (T/N ratio > 2) from other malignancies (GBMs and metastases) when patients were not on corticosteroids. FDG uptake could be influenced by cumulative doses of corticosteroid before a PET scan, a point that should always be considered. A recent meta-analysis [73] on ^18^F-FDG PET and PET/CT in PCNSL, based on 8 retrospectives studies (129 patients), revealed pooled sensitivity and specificity of ^18^F-FDG-PET and PET/CT in the diagnosis of PCNSL of 0.88 and 0.86, respectively.

Okada et al. [69] suggested higher FDG-PET SUVmax and higher ΔSUVmax (ratio of SUVmax in the late and early phase) on MET-PET for PCNSL compared with GBM.

*Take Home Message*. SUVmax is influenced by different factors, in particular the sensitivity of the PET scanner. Therefore, particular attention must be paid when the values of this parameter are imported from literature into own clinical routine.

### 4.2. PCNSL versus TDL

Schiepers et al. [74] reported that active lesions in acute multiple sclerosis are hypermetabolic, while chronic lesions are hypometabolic: thus, glucose uptake could be a marker of the temporal stage of a plaque. Takenaka et al. [75] studied 6 cases of TDLs and found a mean FDG T/N ratio similar to the glucose metabolism of normal cortex and lower than those of GBMs. The same authors described a lower MET uptake in TDL than in glial malignancies. Padma et al. [76] reported low glucose uptake and prominent methionine uptake in a case of TDL: the authors hypothesized that MET uptake in the lesion was related to inflammation and blood-brain barrier disruption. Maffione et al. [77] described moderate focal glucose uptake in two TDLs (SUVmax 6.9 in both cases). Overall, studies comparing ^18^F-FDG PET and MET-PET findings in PCNSL and TDLs are lacking. Patients with demyelinating diseases can show diffuse cortical hypometabolism due to chronic white matter damage [78, 79], especially in patients with subacute/chronic neurological impairment and small white matter lesions. However, cortical hypometabolism does not exclude a diagnosis of lymphoma, as it can be an expression of a cortical disconnection due to white matter lesions (tumoral or not). The use of MET-PET could more precisely indicate the presence of a tumoral lesion [80].

*Take Home Message*. In case of negative or doubtful results at 1 hour ^18^F-FDG postinjection images, it is advisable to repeat a delayed PET acquisition at 4 hours after injection. In some cases, this procedure allows an improvement in the visual analysis.

### 4.3. PCNSL versus Infectious and Granulomatous Diseases

In patients with acquired immunodeficiency syndrome (AIDS), ^18^F-FDG uptake can be used to distinguish between cerebral PCNSL (highly metabolic lesions) and toxoplasmosis (hypometabolic lesions) [81].

Neurosarcoidosis can be hypermetabolic on brain ^18^F-FDG-PET [82]. In suspected neurosarcoidosis, whole-body FDG-PET can help in localizing granulomatous lesions, which can be a target for biopsy [82]. MET-PET yields similar results. Kawai et al. [66] described a series of nontumoral lesions with moderate MET uptake, including brain abscesses. Ng et al. [51] demonstrated MET uptake and high FDG uptake in multiple brain lesions due to neurosarcoidosis. These results are not surprising, since MET uptake can be increased as a result of increased density of inflammatory cells and disruption of the blood-brain barrier (BBB).

**Figure 4:**
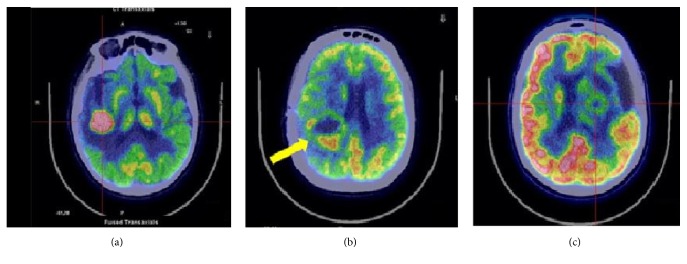
Axial ^18^F-FDG brain imaging. PCNSL (a), glioblastoma multiforme (b), and abscess (c).

*Take Home Message*. ^18^-FDG-PET should be performed, together with MRI spectroscopy and perfusion MRI, when suspecting a PCNSL prior to steroid treatment and biopsy. To date, the major limitation of ^18^F-FDG-PET is the difficulty to diagnose PCNSLs with atypical radiological findings (disseminated or nonenhancing lesions) [Figure 4]. Further studies are needed to determine the diagnostic value of MET uptake and the usefulness of combining MET with FDG-PET. Nonetheless, the ability of MET-PET to detect PCNSL in disseminated or cortical lesions suggest a role in the diagnosis of suspected PCNSL with atypical findings.

## 5. PCNSL Diagnosis: Role of CSF

A recent review on the accuracy of flow cytometry (FCM) and cytomorphology (CM) in the diagnosis of meningeal involvement from lymphoid neoplasms [83], based on 27 studies, demonstrated a great heterogeneity: positive results with both FCM and CM range from 0.3% to 42.9% among studies. Samples with positive FCM but negative CM are reported by 89% of the studies, while samples with positive CM and negative FCM are found in 48%. In a study by Schroers et al. [84] regarding a cohort of PCNSL patients, 23.3% were positive for CSF lymphoma cells with flow cytometry in contrast to 13.3% patients with positive cytopathology. It must be noted that CSF cells are particularly fragile and must be analyzed in the hours following the examination or be put in a stabilizing suspension. Overall, one can suggest employing flow cytometry along with conventional cytology to increase the number of positive CSF cells. Another method to detect mature B lymphoid cells is PCR testing for IgH gene rearrangements, which does not require intact cells [85].

Studies regarding the diagnostic role of different CSF markers (interleukins, chemokines, and receptors) and miRNAs are ongoing. Interleukin-10 (IL-10) and its receptors are expressed in PCNSL and act as a growth factor for B lymphocytes, while IL-6 is related to lymphoid cells growth and immunity regulation. Sasayama et al. [86] demonstrated that CSF IL-10 and IL-6 levels are significantly higher in PCNSLs than in other brain tumors: at an IL-10 cut-off level of 9.5 pg/mL, the sensitivity and specificity were 71.0% and 100%, respectively. Nguyen-Them et al. [87] reported that IL-10 CSF concentration can distinguish PCNSL from other neurologic diseases with sensitivity of 88.6% and specificity of 88.9% with a cut-off of 4 pg/ml, while Song et al. [88] reported diagnostic sensitivity and specificity of 95.5% and 96.1%, respectively, when IL-10 levels cut-off was set at 8.2 pg/ml.

Osteopontin (OPN) is another proinflammatory cytokine involved in immune cell activation and B-cell migration and proliferation. Strehlow et al. [89] demonstrated that CSF OPN levels in PCNSL patients are significantly higher (> 620 ng/mL) than those in patients with inflammatory CNS disease and GBM or in healthy controls. Similarly, Viaccoz et al. [90] reported that CSF neopterin (a marker of neuroinflammation) levels are significantly higher in patients with PCNSL than in those with other brain tumors, pseudotumoral inflammatory brain lesions, or nontumefactive inflammatory CNS disorders, with 96% sensitivity and 93% specificity for the diagnosis of PCNSL.

### 5.1. Take Home Message

Combined “classical” CSF analysis (i.e., CM and CFM) increases the diagnostic accuracy for PCNSL, but a role for CSF markers of B lymphoid cell proliferation is emerging. These new biomarkers could improve the differential diagnosis of PCNSL but need to be validated and standardized before a routine application in clinical practice takes place.

## 6. PCNSL Diagnosis: Role of Ocular Examinations

When PCNSL is suspected, ocular evaluation including fundoscopy and slit lamp examination should be performed. Ocular involvement must be confirmed by vitreous biopsy, and a positive result in a context of brain MRI suspicious for PCNSL can be diagnostic and avoid brain biopsy. A positive cytology is obtained in 50% of cases; as for CSF, immunophenotyping and detection of IgH or T-cell receptor rearrangements by PCR analysis indicating monoclonality are helpful tools for diagnosis. Moreover, high levels of IL-10 and/or high IL-10/IL6 ratio in ocular fluids are strongly suggestive of B-cell lymphomatous uveitis [9]. Fluorescein angiography may be useful for lymphomatous involvement of the retina.

### 6.1. Take Home Message

A cytological confirmation of ocular involvement should be performed whenever feasible.

## 7. PCNSL Diagnosis: Role of Baseline Cognitive Assessment

Cognitive symptoms are critical domains to assess in PCNSL patients, since they can prevail at diagnosis. Neuropsychological baseline testing is fundamental to allow a better interpretation of cognitive decline following therapy and in follow-up phases. Guidelines from Correa et al. [91] outlined the importance of standardization of neuropsychological evaluation in PCNSL: a battery of tests to assess attention, executive function, verbal memory, motor function (domains frequently impaired in PCNSL patients), quality of life, and premorbid IQ was proposed.

Cognitive assessment may sometimes help in distinguishing PCNSL from other nonneoplastic diseases (e.g., Creutzfeldt-Jakob disease, autoimmune encephalitis, paraneoplastic encephalitis, and infectious conditions): in PCNSL patients, memory impairment is more marked than in other rapidly progressive dementias (RPDs), while neurological signs, such as myoclonus and parkinsonism, are very rare [92].

### 7.1. Take Home Message

Cognitive tests should be performed in PCNSL patients before any antineoplastic therapy.

## 8. PCNSL Prognostication: Role of MRI and PET

Recent studies suggest that some parameters of advanced MRI can predict patients' prognosis and tumor responsiveness to methotrexate-based chemotherapy. Zhang et al. [93] investigated ADC as a prognostic indicator in 28 patients with PCNSL receiving high-dose methotrexate-based chemotherapy: patients with higher ADC 5% (ADC at 5% percentile) showed significantly longer progression-free survival (PFS). Similarly, Barajas et al. [94] showed an inverse correlation between cellular density and ADC measurement: in particular, ADC 25% values (ADC at 25% percentile) less than the median value of 692 (“low ADC group”) were associated with significantly shorter progression-free survival (PFS) and overall survival (OS).

Wieduwilt et al. [95] reported similar results, demonstrating shorter PFS and OS in patients with lower ADCmin values (less than 384 X10^−6^) prior to immunochemotherapy. Valles et al. [96] reported that PCNSL patients with low ADCmin or low rCBV values have worse PFS and OS and hypothesized that low tumor rCBV in PCNSL could express a lack of tumor angiogenesis and a decrease in the number of vessels able to deliver intravenous methotrexate to the tumor. In line with these findings, Chung et al. [97] showed that the pattern of DCE-MRI could be a marker for predicting complete response (CR) and longer PFS following chemotherapy, with a strong association between “diffuse” DCE-MRI pattern and CR. Regarding ^18^F-FDG-PET, Kawai et al. [98] demonstrated that pretreatment ^18^F-FDG uptake can predict survival in PCNSL patients: the median survival time of patients with low-to-moderate ^18^F-FDG uptake (SUVmax < 12) was significantly longer (26 months) than that of patients with high ^18^F-FDG uptake (SUVmax ≥ 12; 12 months).

### 8.1. Take Home Message

Prognostication by MRI and PET is thus far limited to clinical trials. Nonetheless, a correlation between lower ADC (reflecting high tumor cellularity), lower rCBV (reflecting poor vascularity), higher FDG metabolism, and relatively poorer response to treatment can be hypothesized.

## 9. PCNSL Prognostication: Role of CSF

CSF IL-10 levels decrease after PCNSL treatment [86, 87] and increase at time of relapse [86]. High baseline and posttreatment IL-10 levels have been associated with poor PFS [86–88]. Moreover, CSF OPN levels have been associated with shorter PFS and OS [89]. Other potential biomarkers of PCNSL, related to disease response to therapy, include CSF levels of transmembrane activator and CAML interactor (TACI), soluble CD19 [99], antithrombin III [100], free immunoglobulin light chains [101], CXCL13 [102], and miRNAs. With regard to the latter, Baraniskin et al. [103] demonstrated the ability of CSF miR-21, miR-19b, and miR-92 levels to differentiate PCNSL from other nonneoplastic neurological disorders and correlate with different phases of PCNSL disease. No role was demonstrated for serum miRNA levels.

### 9.1. Take Home Messages

Prognostication by CSF markers is thus far limited to clinical trials.

## 10. PCNSL Treatment: Monitoring of the Disease

Standard treatment of PCNSL includes 2 phases: induction and consolidation. Induction strategies usually consist in high-dose methotrexate- (HD-MTX-) based polychemotherapy: combination of HD-MTX with other CT agents could improve response compared with HD-MTX alone. Drugs found to be useful and safe when combined with HD-MTX are cytarabine, lomustine, procarbazine, vinca alkaloids, temozolomide, and thiotepa [104]. HD-MTX-based polychemotherapy can be associated with the administration of rituximab (RTX), an anti-CD-20 monoclonal antibody [4, 105].

Induction phase is followed by consolidation treatments, such as whole-brain radiotherapy (WBRT), high-dose myeloablative chemotherapy supported by autologous stem-cell transplantation (HDC-ASCT), and nonmyeloablative chemotherapy. WBRT was for long time used with doses between 40 and 45 Gy in 20–25 fractions, but these doses were associated with important cognitive dysfunctions: for this reason, reduced-dose (RD) WBRT (23.4 Gy) is commonly used. A recent phase III study by Thiel et al. [106] suggested that consolidation with RD-WBRT can improve patients' PFS but not OS. On these bases and considering the risk of neurotoxicity related to RT, consolidation strategies to minimize neurotoxicity have been proposed, such as avoiding WBRT in patients with CR after chemotherapy, lower doses of WBRT, and replacing consolidation radiotherapy with other treatments such as ASCT [107, 108].

A recent International Extranodal Lymphoma Study Group-32 (IELSG32) trial proposed the regimen MATRix (a combination of HD-MTX, HD-ARAC [cytarabine] thiotepa, and rituximab) as new standard chemoimmunotherapy [109]. Treatment regimens based on CT (i.e., HD-MTX alone or associated with other chemotherapeutic agents such as temozolomide) have been suggested as a reasonable approach for elderly patients with good PS and renal function [110–112].

Patients with recurrent/refractory disease typically have poor outcomes, with response rates ranging between 30 and 60% and PFS ranging between 2 and 6 months. The key points in relapsed disease are MTX sensitivity and performance status of the patient. In cases with MTX-sensitive relapse, additional MTX cycles must be administered to achieve maximal cytoreduction (6–8 cycles). This must be followed by dose-intensive CT consolidation in “fit patients,” CNS penetrant agents such as thiotepa, or carmustine-based regimens. Topotecan, temozolomide, pemetrexed, bendamustine, PCV, ifosfamide-etoposide-based regimen, and cisplatin-cytarabine-based regimens are feasible choices for salvage treatment. However, a standard of care thus does not exist so far. The most appropriate salvage treatment should be chosen on the basis of patient's age, PS, comorbidities, site of relapse, previous therapy, and duration of previous response and expected side effects of salvage drugs. HDCT with ASCT is an option in selected relapsed/refractory cases, but there is evidence of superiority of this strategy to chemoradiotherapy [4].

New targeted agents, such as lenalidomide and ibrutinib, are showing preliminary interesting results in relapsed CNS lymphoma [113, 114]. Other promising therapeutic agents are the “check-point inhibitors” of PD-1/PD-L1 pathway, which play a crucial role in tumor immunology [115]. PD-1/PD-L1 expression has been recently detected in 42% of the lymphomas in a large cohort of 10.187 PCNSL samples [116].

## 11. MRI and PET: What to Value and When

The International Primary CNS Lymphoma Collaborative Group defined in 2005 the guidelines for evaluating PCNSL response to treatments based on the evaluation of gadolinium enhancement on MRI, use of corticosteroids, eye examination, and CSF cytology. Combining these elements, patients' response to therapy can be defined as complete response (CR), unconfirmed complete response (uCR), partial response (PR), stable disease (SD), or progressive disease (PD) [117].

Patients enrolled into clinical trials should be assessed after the completion of therapy by brain imaging at a minimum of 3 months for 2 years and then every 6 months for 3 years and yearly for at least 5 years. A recent study by Fossard et al. [118] aimed to define the best follow-up strategy for PCNSL patients after first-line therapy in clinical practice. 125 PCNSL patients, who achieved CR after first-line therapy, started the follow-up and the timing of planned visits and MRI was defined by each center. Overall, serial imaging detected relapses in a minority of asymptomatic patients only. No differences in patient outcome between symptomatic and asymptomatic relapses were seen. Tabouret et al. [119] investigated the prognostic value of conventional MRI characteristic and defined tumor responsiveness to chemotherapy. In a cohort of 85 PCNSLs, infratentorial location was associated with shorter OS and larger enhancing tumor volume correlated with poor PFS; moreover, the percentage of decrease in T1 enhancement between baseline and first MRI evaluation correlated with OS. These authors also described at baseline T2-FLAIR hyperintense lesions distant from the enhancing tumor in 26% of patients: 89% of these lesions decreased after chemotherapy, and in 50% of patients relapses originated from these areas, reinforcing the hypothesis of their neoplastic nature. These data underline the difficulty of interpretation, especially after treatment, of T2-FLAIR hyperintense nonenhancing alterations, which can be variably attributed to vascular damage, leukoencephalopathy related to MTX or radiotherapy, or neoplastic tissue.

Advanced MRI techniques and FDG-PET or MET-PET could improve the prediction of response, progression, and outcome. A reduction of ADC values after chemotherapy correlated with methotrexate responsiveness [93]. Palmedo et al. [120] showed that FDG-PET was able to predict complete remission or tumor recurrence after chemotherapy in 8 patients affected by PCNSL. Jo et al. [121] showed that patients with negative FDG-PET after chemotherapy had significantly longer progression-free survival, but not overall survival, compared to the group with positive posttreatment PET. Conversely, Mercadal et al. [122] detected a difference in overall survival between negative and positive posttreatment FDG-PET patients (100% versus 37.5%, resp., *p* = 0.045). Some authors have hypothized that the reduction of ^18^F-FDG uptake after the first cycles of chemotherapy could precede the reduction of tumor size on MRI [98]. Thus, the combination of ^18^F-FDG-PET and MRI could improve the evaluation of treatment response [123] and the appreciation of residual MRI lesions during follow-up examinations. [122]. MET-PET could be useful for an early delineation of response to systemic chemotherapy or radiotherapy [124].

In conclusion, FDG-PET can be used to evaluate treatment response of PCNSL at a very early stage and predict a more aggressive disease course. During long-term follow-up, FDG-PET findings could anticipate the diagnosis of PCNSL recurrence, even if this hypothesis should be demonstrated in prospective studies with higher number of patients. MET-PET may have a role in identifying residual tumor that is difficult to detect by MRI owing to the effects of previous radiotherapy or surgery [Table 1].

### 11.1. Take Home Message

In the daily clinical practice, monitoring of response/progression with enhanced MRI is mandatory, while advanced MRI techniques and PET imaging are confined to clinical trials.

## 12. Cognitive Monitoring

Serial monitoring of cognitive functions is important for two reasons. First, cognitive impairment can be a marker of recurrence. Fossard et al. [118] reported that 80% of PCNSL relapses were not detected by MRI examinations but on the basis of neurological symptoms, in particular cognitive impairment in 43% of patients. Second, cognitive follow-up is crucial to assess the long-term effects of treatments, in particular central neurotoxicity. Clinically, neurotoxicity presents as a progressive cognitive impairment, which can lead to dementia, and develops after a variable delay from the end of treatment. Cognitive dysfunctions can parallel or follow motor and autonomic symptoms. Clinical features mimic diseases, such as Binswanger dementia or normal pressure hydrocephalus [125]. Risk factors for neurotoxicity include older age, comorbidities, leptomeningeal disease, WBRT, and aggressive chemotherapy regimens. Several chemotherapeutic agents, particularly HD-MTX and high-dose cytarabine, have been shown to cause periventricular white matter abnormalities [126]. Combined regimens, including WBRT, display a higher percentage of neurotoxicity when compared to chemotherapy alone, with an incidence of neurotoxicity ranging from 8% to 50% of patients [126–132]. Combined modality treatments, including RT, are associated with cognitive impairment even in patients below 60 years of age [133], but the frequency is higher above 60 years of age. For this reason, many authors suggest to defer WBRT at recurrence in older patients [134–136]. Encouraging results are coming from trials on HDC-ASCT, with no significant neurotoxicity and even improved cognitive functions and/or quality of life [108, 125].

The possibility to derive information on treatments neurotoxicity depends on cognitive testing of patients in remission, since in cases of PCNSL progression the cognitive decline depends on the disease itself [91].

### 12.1. Take Home Message

In daily clinical practice, cognitive testing should be encouraged to better define the cognitive profile in the different scenarios (persistent response versus disease recurrence).

## 13. Conclusions

The knowledge of neuroimaging and biological markers of PCNSL is rapidly growing. Thus, it is conceivable that in future years the scenario of PCNSL management will change, moving to a better disease-profiling and “tailored-on-patient” therapies. Cooperation and scientific dialogue between different medical professionals (neurologists, neuroradiologists, nuclear medicine physicians, hematologists, oncologists, neurosurgeons, ophthalmologists, pathologists, and psychologists) are essential to improve diagnosis, prognosis, treatment strategies, and outcomes of such a complex disease in both clinical trials and everyday clinical activity.

## Figures and Tables

**Table 1:** Application of advanced brain imaging in PCNSL diagnosis and monitoring.

	**Diagnosis**	**Monitoring**
**MRI DWI/ADC**	(i) Marked reduction of ADC values in PCNSL in comparison to HGGs and TDLs	(i) No clear data

**MRI spectroscopy**	(i) Higher lip/Cr ratios in PCNSL in comparison to nonnecrotic areas of HGGs (ii) Similar MRS patterns between PCNSL and TDLs	(i) Useful for metabolic information about T2-FLAIR hyperintense lesions

**MRI perfusion**	(i) Lower rCBV values in comparison to GBMs	(i) No clear data

^**18**^ **F-FDG-PET**	(i) Homogeneous FDG uptake in PCNSL versus inhomogeneous uptake in GBMs or metastases	(i) Useful for assessment of treatment response (ii) Useful for metabolic information about T2-FLAIR hyperintense lesions (iii) Potential role for postchemotherapy FDG-PET in predicting PFS/OS

**MET-PET**	(i) Limited experience (ii) Potential role for the identification of disseminated or nonenhancing lesions	(i) Useful for assessment of treatment response (ii) Potential role for tumor delineation after CT and RT; potential role for
		detection of residual tumor
